# Mayaro virus, a potential threat for Europe: vector competence of autochthonous vector species

**DOI:** 10.1186/s13071-024-06293-7

**Published:** 2024-05-04

**Authors:** Marco Brustolin, Koen Bartholomeeusen, Tatiana Rezende, Kevin K. Ariën, Ruth Müller

**Affiliations:** 1 Department of Biomedical Sciences, Entomology Unit, Institute of Tropical Medicine Antwerp, Antwerp, Belgium; 2 Department of Biomedical Sciences, Virology Unit, Institute of Tropical Medicine Antwerp, Antwerp, Belgium; 3 Present Address: Institute René Rachou, Fundação Oswaldo Cruz (FIOCRUZ), Belo Horizonte, Brazil

**Keywords:** Mayaro virus, *Aedesalbopictus*, *Anophelesatroparvus*, *Culexpipiens*, Vector competence, Europe

## Abstract

**Background:**

Mayaro virus (MAYV) is an emerging alphavirus, primarily transmitted by the mosquito Haemagogus *janthinomys* in Central and South America. However, recent studies have shown that *Aedes* *aegypti*, *Aedes* *albopictus* and various *Anopheles* mosquitoes can also transmit the virus under laboratory conditions. MAYV causes sporadic outbreaks across the South American region, particularly in areas near forests. Recently, cases have been reported in European and North American travelers returning from endemic areas, raising concerns about potential introductions into new regions. This study aims to assess the vector competence of three potential vectors for MAYV present in Europe.

**Methods:**

*Aedes* *albopictus* from Italy, *Anopheles* *atroparvus* from Spain and *Culex* *pipiens* biotype *molestus* from Belgium were exposed to MAYV and maintained under controlled environmental conditions. Saliva was collected through a salivation assay at 7 and 14 days post-infection (dpi), followed by vector dissection. Viral titers were determined using focus forming assays, and infection rates, dissemination rates, and transmission efficiency were calculated.

**Results:**

Results indicate that *Ae.* *albopictus* and *An.* *atroparvus* from Italy and Spain, respectively, are competent vectors for MAYV, with transmission possible starting from 7 dpi under laboratory conditions. In contrast, *Cx.* *pipiens* bioform *molestus* was unable to support MAYV infection, indicating its inability to contribute to the transmission cycle.

**Conclusions:**

In the event of accidental MAYV introduction in European territories, autochthonous outbreaks could potentially be sustained by two European species: *Ae.* *albopictus* and *An.* *atroparvus*. Entomological surveillance should also consider certain *Anopheles* species when monitoring MAYV transmission.

**Graphical abstract:**

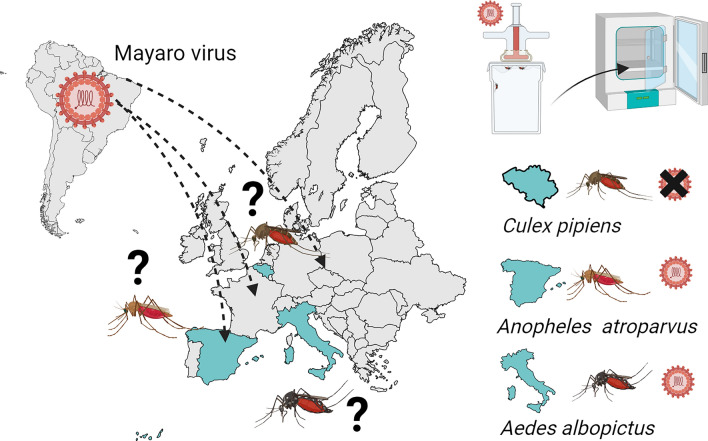

**Supplementary Information:**

The online version contains supplementary material available at 10.1186/s13071-024-06293-7.

## Background

The Mayaro virus (MAYV), classified within the Alphavirus genus of the *Togaviridae* family, was initially isolated from febrile workers in Mayaro County, Trinidad, in 1954 [1]. With a single-stranded positive-sense RNA genome of approximately 11.7 kb, MAYV is phylogenetically classified into three genotypes: D, L, and N. Genotype D spreads across multiple South American countries, while genotype L remains confined to Brazil [2]. The less prevalent genotype N emerged in Peru during 2010 [3]. MAYV fever manifests in humans as a mild to severe self-limited illness, characterized by abrupt fever, arthralgia/arthritis and maculopapular rash. Additional symptoms may include headache, myalgia, retro-orbital pain, vomiting and diarrhea. This acute incapacitating disease, akin to chikungunya virus (CHIKV;* Togaviridae*, Alphavirus) fever, often leads to prolonged arthralgia in over 50% of cases [4, 5]. The primary vector for MAYV is the canopy-dwelling mosquito *Haemagogus* *janthinomys* (Dyar, 1921) [6]. Several vector competence studies indicate that the yellow fever mosquito *Aedes* *aegypti* (Linnaeus, 1762) and the Asian tiger mosquito *Aedes* *albopictus* (Skuse, 1895) are competent vectors of MAYV [7, 8]. In addition, we recently demonstrated that at least five different *Anopheles* species are competent vectors for MAYV under laboratory conditions [9, 10], highlighting MAYV’s considerable plasticity in terms of permissive vector species. Over time, MAYV has triggered sporadic outbreaks and localized epidemics, especially in communities living and working in close proximity to the Amazon forest. In the last decade, however, the number of outbreaks has increased, likely due to the increase of anthropogenic activities, including landscape fragmentation [11]. As a consequence, the Pan American Health Organization (PAHO) suggested increasing the level of awareness and surveillance [12]. Given the expanding geographical area and the rising number of local MAYV cases, it is reasonable to anticipate an increase in the number of imported cases, as travelers will have a higher probability of becoming infected during their stay abroad. Several cases of MAYV-infected travelers have already been reported in Europe, spanning countries such as the Netherlands [13], Germany [14], France [15] and Switzerland [16], and highlighting the potential of this pathogen to spread to new regions. This phenomenon is similar to what already happened with other major arboviruses, such as CHIKV [17, 18] and dengue virus (DENV; *Flaviviridae*, Flavivirus) [19, 20] in Europe. The last imported case of Mayaro was in France during 2016 [21]. Since then, *Ae.* *albopictus*, a competent vector species for MAYV, has largely spread across Europe, reaching the Central-North area of Europe [22], thereby increasing the risk of local transmission of several arboviruses such as CHIKV, DENV, Zika virus (ZIKV; *Flaviviridae*, Flavivirus) and potentially MAYV.

Despite the call to attention regarding MAYV by the PAHO, nothing is known about the vector competence of European mosquito populations for this emerging arbovirus. Therefore, it is crucial to investigate the capacity of autochthonous species to sustain the transmission cycle of this emerging pathogen, in case of its introduction to the European continent.

Vector-borne diseases are often characterized by complex biological cycles, in which multiple mosquito species can transmit the pathogen to different hosts (human and/or animals). In this context, it is of the utmost importance to determine the role of each potential vector species, especially in naive regions [23]. To address this knowledge gap, we investigated the vector competence of three European mosquito species for MAYV under laboratory condition: *Ae.* *albopictus*, *Anopheles* *atroparvus* (van Thiel, 1927), and *Culex* *pipiens* bioform *molestus* (Forskål, 1775) (hereafter referred to as *Cx.* *pipiens*). These particular mosquito species were selected for this study based on their feeding habits, geographical distribution and expected significance as potential vectors. All three species are widely dispersed throughout Europe, occupying diverse ecological niches, including urban areas (*Ae.* *albopictus* and *Cx.* *pipiens*) and peri-urban and rural regions (*An.* *atroparvus*). *Aedes* *albopictus* and *Cx.* *pipiens* exhibit an aggressive feeding behavior, primarily biting humans and occasionally other domestic mammals such as cats, dogs and avian species [24, 25], while *An.* *atroparvus* displays opportunistic feeding behaviors, readily biting humans or wild and domestic animals such as deer, goats, horses and bovines [26, 27]. In Europe, *Ae.* *albopictus* and *Cx.* *pipiens* are prominent vector species, with *Ae.* *albopictus* serving as a primary vector for ZIKV, CHIKV and DENV, while *Cx.* *pipiens* acts as a primary vector for West Nile virus and Usutu virus [28, 29]. Although *An.* *atroparvus* was identified as a key vector of malaria during the twentieth century [30], its potential for transmitting any arbovirus was previously unknown.

Our results demonstrate for the first time that European populations of *Ae.* *albopictus* and *An.* *atroparvus* are capable of sustaining the replication cycle of MAYV and of efficiently transmitting the virus through saliva starting from day 7 post feeding. Conversely, *Cx.* *pipiens* was found to be refractory to MAYV infection. Our findings emphasize that surveillance plans and entomological control measures should be adapted to include *Anopheles* species which may contribute to the transmission cycle of MAYV and potentially other exotic arthropod-borne viruses.

## Methods

Three mosquito species were investigated for their potential MAYV vector competence. *Anopheles* *atroparvus* (strain Ebre delta) was originally collected in Catalonia, Spain in 2020 [30]; *Cx.* *pipiens* was repeatedly collected in Antwerp, Belgium during summer 2020 [31]; and *Ae.* *albopictus* was collected in Terni, Italy, in 2021 [32]. Following field collections, laboratory colonies were established at the Merian Insectarium of the Institute of Tropical Medicine Antwerp (Belgium). Mosquito colonies were reared and maintained under the following environmental conditions: (i) *Ae.* *albopictus* and *An.* *atroparvus:*  27 °C and a light/dark cycle of 11.5/11.5 h; (ii) *Cx.* *pipiens*:  23 °C and a light/dark cycle of 15.5/7.5 h. Two crepuscular cycles of 30 m each in between the day and night cycles were add to simulate dusk and dawn. Colonies were kept at 80% relative humidity in 30 × 30 × 30-cm cages (BugDorm-1 type; Megaview, Taichung, Taiwan) with access to 10% glucose solution + 0.1% of methyl paraben (Sigma-Aldrich, St. Louis, MO, USA). During larvae stages, *Aedes* and *Culex* larvae were fed on Koi mini sticks (Tetra, Melle, Germany) and *Anopheles* larvae on Novo Fect (JBL, Neuhoken, Germany).

### Molecular identification of *Culex pipiens* bioform

The molecular identification of the bioform *Cx.* *pipiens* form *pipiens* versus *Cx.* *pipiens* form *molestus,* of all specimens used was performed by PCR, targeting the CQ11 microsatellite region. Briefly, mosquito DNA was extracted using the DNeasy Blood & Tissue Kit (Qiagen, Hilden, Germany) following the manufacturer’s recommendation. Extracted samples were used as template and amplified using the forward primer CQ11F2 and the reverse primers pipCQ11R and molCQ11R. The primers, reagents and conditions were previously described [33].

### Virus and cells

Mayaro virus strain BeAn 343102 (NR-49909; BEI Resources, Manassas, VA, USA) was used for the experimental infections. Strain BeAn 343102 belongs to genotype D and was originally isolated from a monkey in Para (Brazil) in 1978. The virus was propagated on Vero E6 cells (CRL-1586; ATCC, Manassas, VA, USA), and the stock solution was aliquoted and stored at − 80 °C until used. Viral stock and sample titers were determined using the focus forming assay (FFA) with Vero E6 cells.

### Vector competence assay

**Fig. 1:**
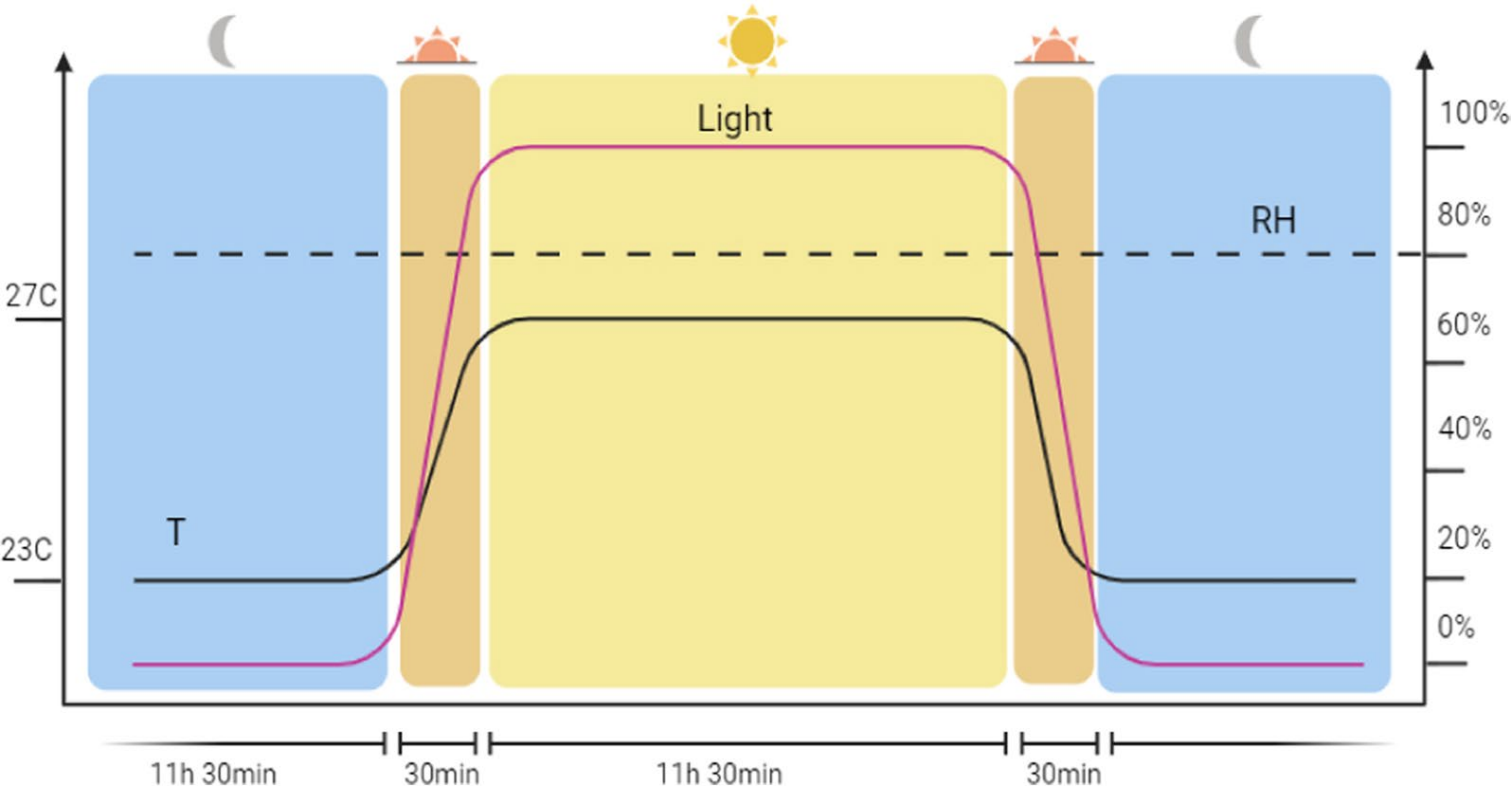
Environmental conditions for the vector competence assay. These conditions were applied starting from − 1 day post infection (transfer of mosquitoes to arthropod containment level 3 unit and acclimatization to a light/dark cycle of 11.5/11.5 h (shown in yellow and blue, respectively, on the graph) separated by two periods of 30 min to simulate dusk and dawn (in orange). Temperature (T, black line) was set at 23 °C during the nighttime and 27 °C during the daytime. During dusk and dawn (duration of 30 min each), temperature changed at a rate of 1 °C/5 min and light intensity (purple line) changed at a rate of 20%/5 min. Relative humidity (RH, dotted line) was set constant to 80%. Figure was created with BioRender.com

Female mosquitoes aged 5–7 days were used to perform the vector competence studies. Approximately 80 females of each species were caged inside a 450-ml cardboard cup covered with mesh screen and the cup transferred to inside the Finlay arthropod containment level 3 facility at 1 day before the experimental infection to allow acclimatization. Mosquitoes were deprived of sugar 18 h before the start of the experiment to stimulate feeding. During the feeding, mosquitoes were exposed to MAYV-spiked human blood (final titer of 1 × 10^7^ focus-forming units [FFU]/ml) via the Hemotek feeding system (SP6W1-3; Hemotek Ltd, Blackburn, UK) using a collagen membrane (MEM5; Hemotek Ltd) at 38 °C for 45 min. After the feeding, mosquitoes of each group were anesthetized at 4 °C, and fully engorged females were selected, randomly allotted to two subgroups, housed in clean cardboard cups with access to cotton soaked in 10% glucose solution and stored in climatic cupboards under the following controlled environmental condition: light/dark cycle of 11.5/11.5 h, with two crepuscular cycles of 30 min each in between to simulate dusk and dawn; the mean day and night temperature was 27 °C and 23 °C, respectively. During dawn and dusk, the temperature increased or decreased by 1 °C/5 min (Fig. 1).

**Fig. 2:**
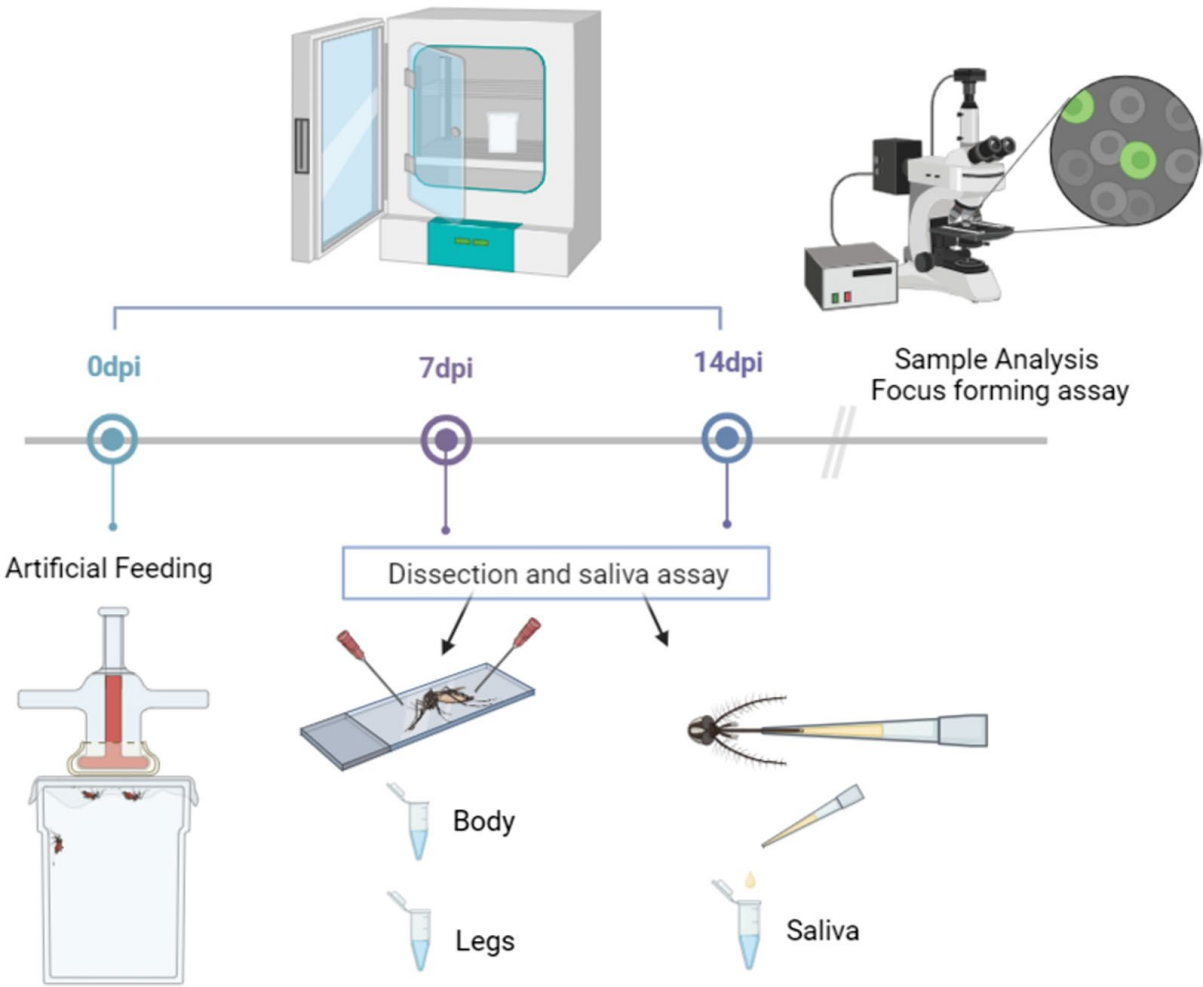
Vector competence assay. Mosquitoes were artificially exposed to infectious blood meals at a final titer of 1 × 10^7^ FFU/ml MAYV, and maintained in a climatic chamber (under the condition shown in Fig. 1). At 7 dpi and 14 dpi, mosquito saliva was collected, and the body and legs of each mosquito were processed individually. The viral titer of all samples (body, legs and saliva) was determined by the focus forming assay and expressed as FFU/ml. dpi, Days post infection; FFU, focus-forming units; MAYV, Mayaro virus. Figure was created with BioRender.com

At 7 and 14 days post infection (7 dpi and 14 dpi, respectively), mosquitoes were anesthetized with triethylamine (Sigma-Aldrich). Their legs were dissected, and immobile mosquitoes were forced to salivate into a 20-µl pipette tip filled with 10 µl of a 1:1 solution of 50% sucrose solution and fetal bovine serum (FBS) for 30 min. After 30 min, the saliva was pipetted into a 2-ml Eppendorf tube containing 90 µl of mosquito medium (20% FBS in Dulbecco’s phosphate-buffered saline [PBS], 50 mg/ml penicillin/streptomycin, 50 mg/ml gentamicin and 2.5 mg/ml fungizone). Each mosquito body and respective legs were placed in separate 2-ml Eppendorf tubes containing 1 ml of mosquito medium and a zinc-plated steel 4.5-mm bead (Fig. 2). Body and leg samples were homogenized at 30 Hz for 2 min using the TissueLyser II sample disruption system (Qiagen) and then centrifuged for 30 s at 11,000 rpm. All samples were stored at − 80 °C for further analysis. Three technical replicates were performed for *Ae.* *albopictus* and *An.* *atroparvus*, and two technical replicates were done for *Cx.* *pipiens*.

### Focus forming assay

To determine vector competence status, we tested the infectious viral load in the body, legs and saliva samples of individual mosquitos using the focus forming assay, as previously described [34]. Briefly, samples were diluted tenfold multiple times, added to a confluent monolayer of Vero E2 cells and incubated for 24 h at 37 °C and 5% CO_2_. Cells were fixed with 4% paraformaldehyde (Sigma-Aldrich) in cold PBS 1×, blocked and permeabilized with blocking buffer (3% bovine serum albumin and 0.05% Tween 20 in cold PBS 1×). Immunolabeling was performed with the monoclonal anti-chikungunya virus E2 envelope glycoprotein clone CHK-48 (cross-reactive with MAYV) (BEI Resources) diluted 1:1000 in blocking buffer. After four washes with cold PBS 1×, the primary antibody was labeled with the Alexa-488 goat anti-mouse immunoglobulin G secondary antibody (Invitrogen, Life Technologies, Thermo Fisher Scientific, Waltham, MA, USA) at a dilution of 1:1000 in blocking buffer, and green fluorescent foci were observed and enumerated with a Leica DMi8 fluorescence microscope (Leica Microsystems, Wetzlar, Germany). Viral titer was expressed as FFU/ml, and infection rate (IR), dissemination rate (DIR) and transmission efficiency (TE) were calculated as following: IR = proportion of mosquitoes with a positive body among the total number of mosquitoes exposed to the infectious blood meal that had been analyzed; DIR = proportion of mosquitoes with positive legs among mosquitoes with a positive body; TE = proportion of mosquitoes with positive saliva among the total number of mosquitoes exposed to infectious blood meal that had been analyzed.

### Statistical analysis

Data obtained were analyzed using GraphPad Prism version 10.0.3 (GraphPad Software, San Diego, CA, USA. Differences in IR, DIR and TE between and within mosquito species and between time points (7 dpi vs 14 dpi) were analyzed by Fisher’s exact test. Two-tailed Mann–Whitney U-tests were used to compare viral titers in the body, legs and saliva between and within mosquito species at different time points. The level of significance was set to *P* ≤ 0.05.

## Results

A total of 69 *Ae.* *albopictus* (31.2% combined feeding rate), 100 *An.* *atroparvus* (81.5% combined feeding rate) and 30 *Cx.* *pipiens* (25.2% combined feeding rate) were analyzed in the vector competence assays. Full details on the total number of mosquito tested per species and per time point, and on the IR, DIR and TE are described in Table 1.

**Table 1:** Infection rate, dissemination rate and transmission efficiency

Mosquito species	NX^a^	NE^a^ (% feeding)	7 dpi				14 dpi			
			*N* tested^a^	IR^b^, *N* (%)	DIR^b^, *N* (%)	TE^b^, *N* (%)	*N* tested^a^	IR^b^, *N* (%)	DIR^b^, *N* (%)	TE^b^, *N* (%)
*Aedes* *albopictus*	237	74 (31.2)	33	30 (90.9)	19 (63.3)	5 (15.2)	36	33 (91.7)	30 (90.9)	2 (5.6)
*Anopheles* *atroparvus*	146	119 (81.5)	56	41 (73.2)	26 (63.4)	3 (5.4)	44	34 (77.2)	32 (94.1)	6 (13.6)
*Culex* *pipiens*	119	30 (25.2)	14	0	0	0	16	0	0	0

*dpi* Days post infection

^a^NX (total number of mosquitoes exposed to infectious blood meal; cumulative across all replicates), NE (total number of fully engorged females and percentage feeding; cumulative across all replicates),* N* (number of mosquitoes analyzed per species and time point). Discrepancies between NE and *N* tested are due to mosquito mortality during the extrinsic period of incubation

^b^IR (Infection rate),DIR (dissemination rate) and TE (transmission efficiency) are reported as the number of positive samples and their percentage (%), as defined in the Methods section

**Fig. 3:**
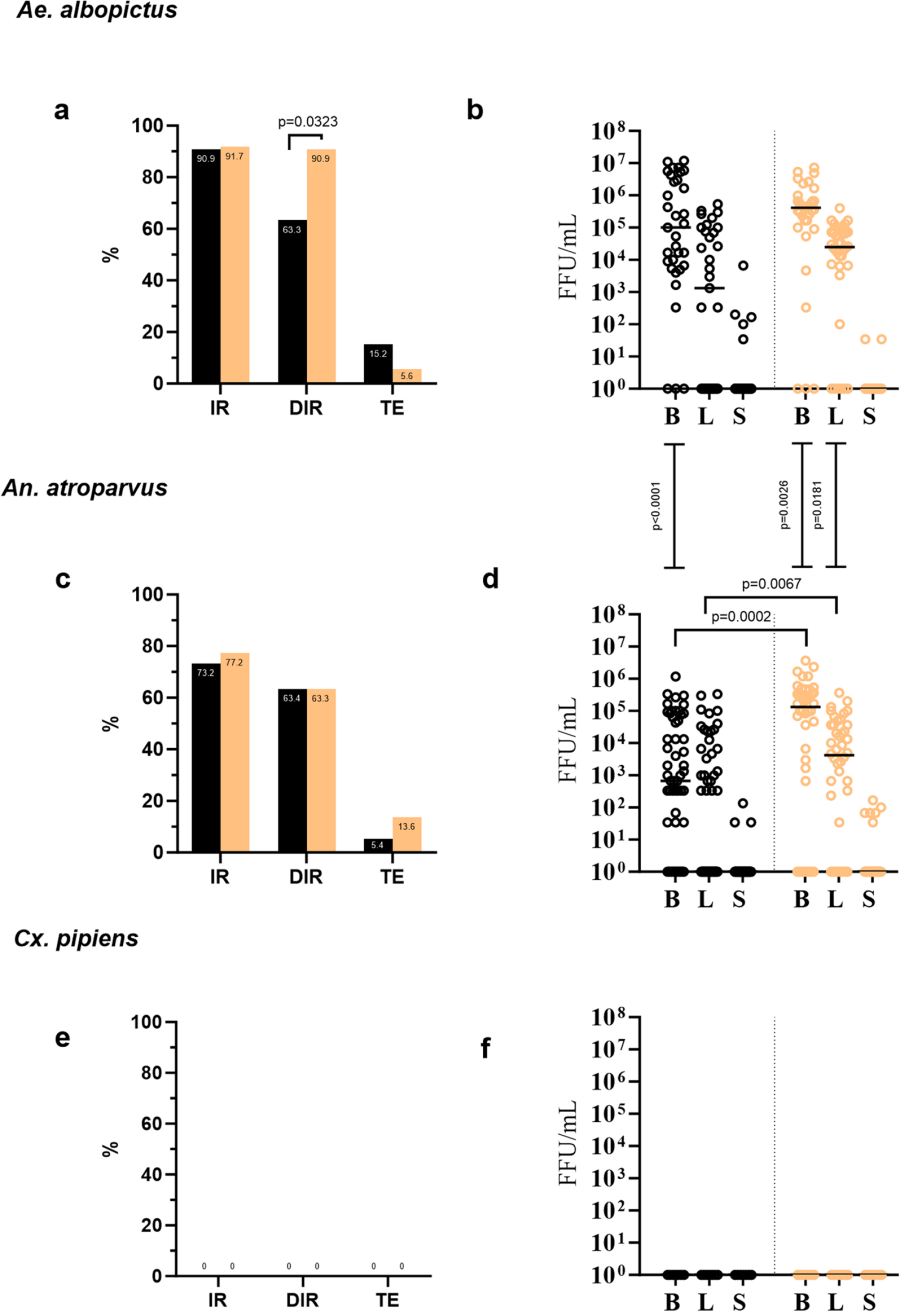
Infection parameters and MAYV viral titer in the body, legs and saliva of mosquitoes. **a**,** c**,** e** Infection rate, dissemination rate and transmission efficiency at 7 dpi (black) and 14 dpi (orange) for *Ae.* *albopictus *(**a**), *An.* *atroparvus* (**c**) and *Cx.* *pipiens* (**e**). **b**,** d**,** f** Viral titer expressed as focus forming units/milliliter (FFU/ml) in the body, legs and saliva of *Ae.* *albopictus *(**b**), *An.* *atroparvus* (**d**) and *Cx.* *pipiens* (**f**) at 7 dpi (black) and 14 dpi (orange). Statistics in **a**, **c**, **e**: Fisher’s exact test between and within mosquito species and between time points. Statistics in **b**, **d**, **f**: two-tailed Mann–Whitney U-tests were used to evaluate significance between and within mosquito species and between time points time points. *P*-value ≤ 0.05 were considered to be significant. B, Body; DIR, dissemination rate; dpi, days post infection; IR, infection rate; L, legs; MAYV, Mayaro virus; S, saliva; TE, transmission efficiency

Among the tested species, *Ae.* *albopictus* and *An.* *atroparvus* proved to be competent vectors, with 15.2% (7 dpi) and 13.6% (14 dpi) of the mosquitoes of these two species showing MAYV in their saliva, respectively (Table 1; Fig. 3a, c, TE). The infection in *Ae.* *albopictus*' bodies remained consistently high throughout the entire experiment, with very similar infection rates between 7 and 14 dpi (90.9% and 91.7%, respectively).

In *Ae.* *albopictus*, there was a statistically significant increase in DIR between the two analyzed time points (63.3% at 7 dpi vs 90.9% at 14 dpi; *P* = 0.0323). No statistically significant variation was found regarding MAYV viral titer in the body, legs or saliva samples between 7 and 14 dpi. *Anopheles* *atroparvus* presented relatively high IR, DIR and TE, with minimal and not significant variations between 7 and 14 dpi (Table 1; Fig. 3c). Contrary to the observations in *Ae.* *albopictus*, viral titer in the bodies and legs of positive mosquitoes increased significantly between the two time points analyzed (Fig. 3d; body, *P* = 0.0002; legs, *P* = 0.0067). Comparative analysis showed no statistical difference in IR, DIR and TE between *Ae.* *albopictus* and *An.* *atroparvus*. However, MAYV viral titer was significantly higher in the body (7 dpi, *P* < 0.0001; 14 dpi, *P* = 0.0026) and legs (14 dpi: *P* = 0.0181) of *Ae.* *albopictus* compared to those of *An.* *atroparvus* (Fig. 3b, d). Conversely to *Ae.* *albopictus* and *An.* *atroparvus*, *Cx.* *pipiens* remained completely refractory to infection, with an IR of 0% (Table 1; Fig. 3e).

Molecular characterization of *Cx.* *pipiens* bioform demonstrated that 86.6% of the mosquitoes tested belongs to bioform *molestus*, with remaining 13.3% identified by molecular characterization as *Cx.* *pipiens* bioform hybrid (Additional file 1: Fig. S1).

## Discussion

To our knowledge, this is the first study describing the vector competence of European mosquito species for MAYV. *Aedes* *albopictus* and *An.* *atroparvus* proved to be competent vectors for MAYV, capable of transmitting the virus starting from 7 dpi under laboratory conditions. In addition, our results showed that the European population of *Ae.* *albopictus* is also capable of transmitting MAYV. *Aedes* *albopictus* is widely distributed throughout Europe. Consequently, it is imperative to test various populations of *Ae.* *albopictus* from Central and Northern Europe to gain a more comprehensive understanding of their competence as vectors for MAYV. Infection, dissemination and transmission results obtained with this European *Ae.* *albopictus* population are comparable to those previously described using a mosquito population from Florida [7], but lower than those reported using a mosquito population from New York [35]. The notable difference is particularly evident for the TE. In these two previous study [7, 35], the authors detected a TE ranging from 30% to 43% at 7 dpi (against 15.2% for the European population) and from 43% to 80% at 14 dpi (against 5.6% for the European population). Since both studies used MAYV strain TRVL-4675 (a genotype D strain), the higher rates obtained in the study of Dieme et al*.* [35] are probably due to (i) different genetic background of the mosquito population; (ii) higher viral titer used for the experiment (8.1 ± 1.1 log10 PFU/ml); and/or (iii) different temperature regime during the extrinsic period of incubation. The European population of *Ae.* *albopictus* in the present study exhibited a higher viral titer of MAYV in the body, legs, and saliva samples than that reported by Wiggins et al*.* for the Florida population [7] (viral titer of MAYV for New York population are not available). The higher viral titer present in the saliva of infected mosquitoes in the European population may indicate a greater transmission potential to a susceptible host, as the viral titer can influence the likelihood of virus transmission.

In the present study, we report for the first time that *An.* *atroparvus* is a competent vector for MAYV; this result is similar to those results previously reported for other *Anopheles* species, including *An. stephensi*, *An.* *gambiae*, *An.* *quadrimaculatus*, *An.* *freebornii* and *An.* *albimanus* [9, 10, 35]. We observed relatively lower values for IR, DIR and TE for *An.* *atroparvus* at 7 dpi, when compared with those of *Ae.* *albopictus* (Table 1; Fig. 3). Although these differences in IR, DIR and TE did not reach statistical significance, they may reflect a lower tropism of MAYV for *An.* *atroparvus*. This hypothesis gains support from a significantly lower MAYV titer found in the bodies and legs of *An.* *atroparvus* compared to those observed in *Ae.* *albopictus*. Our results, together with previously reported findings for other *Anopheles* species, confirm the considerable vector plasticity of MAYV and highlight the necessity to reconsider the role of members of the genus *Anopheles* as a potential source of vector species for arboviruses. *Anopheles* spp. have historically been considered to be only poor vectors of arboviruses. In nature, O'nyong-nyong virus (ONNV; *Togaviridae*, Alphavirus) and Rift Valley fever virus (RVFV; *Phenuiviridae*, Phlebovirus) are the only arboviruses known to be transmitted by *Anopheles* mosquitoes [36–38]. Although several other pathogenic arboviruses and insect-specific viruses have been identified/isolated from *Anopheles* species [39], the role of *Anopheles* in the transmission cycle of the majority of these remains unknown. This lack of information underscores the need for a more in-depth study of the *Anopheles*-arbovirus interaction.

Both *Ae.* *albopictus* and *An.* *atroparvus* are competent vectors for MAYV and can potentially sustain local transmission. However, it is important to note that in the present study, we investigated vector competence, which is one of the parameters used to determine vectorial capacity. Vectorial capacity represents a more complete measure of the potential of a vector population to transmit a pathogen, taking into account factors such as vector density, host availability, and vector survivability. In this context, it is interesting to note that these two species occupy different ecological niches. The distinctive ecological habitats of *Ae.* *albopictus* and *An.* *atroparvus* (urban vs peri-urban), which are increasingly converging due to landscape fragmentation, urban expansion and climate change [11, 40], expand the geographical area for potential MAYV transmission risk. An ecological niche modeling to provide a detailed spatial analysis of MAYV vector dynamics, similar to the modeling previously described for DENV [41], should be conducted for re-thinking vector control programs in the case of local MAYV transmission.

Finally, *Cx.* *pipiens* bioform *molestus* was found to be totally refractory to MAYV infection and cannot contribute to the transmission cycle of this pathogen. Despite the low number of individuals tested, this result is similar to the findings previously described for *Culex* *quinquefasciatus* [10] and suggests a poor vector competence of *Culex* spp. for MAYV. However, more evidence is needed to strengthen this hypothesis, including: (i) replicate vector competence with different populations/bigger number of mosquitoes, and (ii) testing the *Cx.* *pipiens* bioform *pipiens* in order to obtain a better insight into differences in vector competence between bioforms.

Our study demonstrates the capacity of *Ae.* *albopictus* and *An.* *atroparvus* to sustain local transmission of MAYV in the case of its introduction. While these data are of major importance, multiple aspects associated with an autochthonous transmission event remain unsolved. In the present study, we investigated vector competence using one viral strain and a single infectious viral titer in the blood meal. Dose-dependent studies with different viral strains should be performed to fully understand the risk of transmission. Another important aspect to consider is the capacity of MAYV to circulate in different animal hosts. Non-human primates are considered to be the main reservoir animal for MAYV, but evidence of circulation in small mammals and rodents has been reported [42]. To date, few data are available regarding potential animal reservoirs for MAYV, especially in urban and peri-urban contexts. Given the significant variety of vertebrate species normally included within the feeding habits of *An.* *atroparvus* and *Ae.* *albopictus*, a targeted susceptibility study would provide better insights into possible animal hosts in Europe.

## Conclusions

Understanding the vector competence of mosquito species is crucial for controlling and preventing the transmission of viruses by arthropods. Although predicting the emergence of a disease is challenging and can be influenced by multiple factors, our findings indicate that MAYV may poses a health threat in several regions of Europe where *Ae.* *albopictus* and *An.* *atroparvus* are present. Vector competence studies for several autochthonous European species and/or population should be performed. In addition, a study aimed to test the capacity of different local vertebrate to serve as sensible host/reservoir should be performed. Finally, local authorities in Europe should enhance epidemiological and entomological surveillance measures so as to be able to promptly detect the introduction of this viral pathogen.

### Supplementary Information

**Additional file 1: Figure S1.** Molecular characterization of *Cx.* *pipiens*. All mosquitoes used during vector competence assay were molecularly characterized to bioform level. Expected band for *Cx.* *pipiens* biorm *molestus* was 284 bp; the expected band for *Cx. pipiens* bioform *pipiens* was 258–266 bp. The *Cx.* *pipiens* bioform hybrid presents both bands. The figure shows an example of such characterization on a gel electrophoresis. m, *Cx.* *pipiens* biorm *molestus*; h, *Cx.* *pipiens* biorm hybrid; C+, positive control *Cx.* *pipiens* biorm hybrid.

## Data Availability

All data are included as tables and figures within the article.
