# Supplementary material for: Mayaro virus, a potential threat for Europe: vector competence of autochthonous vector species
Source: Parasit Vectors. 2024 May 4;17:200. doi: 10.1186/s13071-024-06293-7 (PMC11071154; doi:10.1186/s13071-024-06293-7)
Supplement: Supplementary file 1 — Additional file 1: Figure S1. Molecular characterization of Cx. pipiens. All mosquitoes used during vector competence assay were molecularly characterized to bioform level. Expected band for Cx. pipiens biorm molestus was 284 bp; the expected band for Cx. pipiens bioform pipiens was 258–266 bp. The Cx. pipiens bioform hybrid presents both bands. The figure shows an example of such characterization on a gel electrophoresis. m, Cx. pipiens biorm molestus; h, Cx. pipiens biorm hybrid; C+, positive control Cx. pipiens biorm hybrid. [file 13071_2024_6293_MOESM1_ESM.docx]

**
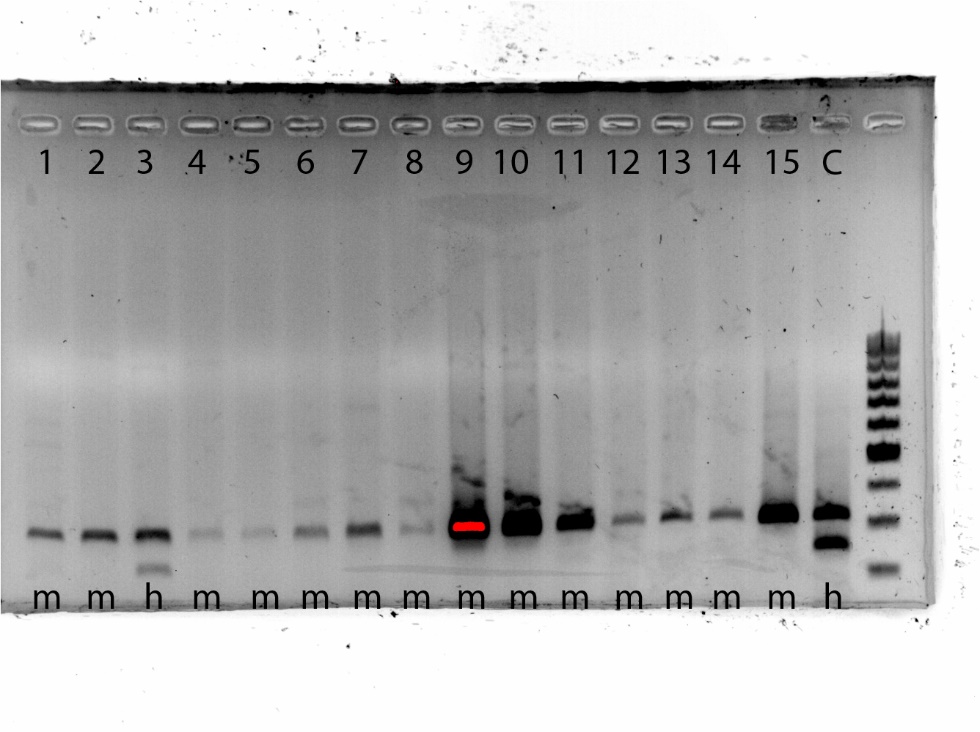
Figure S1: Molecular characterization of *Cx. pipiens*.** All mosquitoes used during vector competence assay has been molecularly characterized to bioform level. Expected band for *Cx. pipiens biorm molestus* = 284bp, Expected band for *Cx.pipiens bioform pipiens* = 258-266. *Cx. pipiens bioform hybrid* presents both bands. The figure shows an example of such characterization on a gel electrophoresis. m= *Cx. pipiens biorm molestus*; h= *Cx. pipiens biorm hybrid*; C+= Positive control *Cx. pipiens biorm hybrid*.
